# Evidence for G-quadruplex-mediated transactivation by the immediate-early 2 protein of human cytomegalovirus

**DOI:** 10.1128/jvi.01467-25

**Published:** 2025-11-25

**Authors:** Shuang Gong, Daegyu Park, Woo-Chang Chung, Jin-Hyun Ahn

**Affiliations:** 1 Department of Microbiology, Sungkyunkwan University School of Medicine 35019, Suwon, Republic of Korea; Dartmouth College Geisel School of Medicine, Hanover, New Hampshire, USA

**Keywords:** G-quadruplex, HCMV, IE2, UL146, transactivation

## Abstract

G-quadruplex (G4) formation in gene promoters regulates promoter activity either positively or negatively. Although G4s have been shown to enhance promoter activation by serving as structural elements that recruit transcription factors, similar information on viral promoters is limited. We recently found that the human cytomegalovirus (HCMV) 86 kDa immediate-early 2 (IE2) protein, which acts as a viral transactivator, binds to a parallel G4 structure in the viral lytic replication origin, *ori*Lyt, playing a role in initiating viral DNA replication. Here, we show that IE2 can transactivate a viral promoter by targeting G4. In HCMV-infected cells, the transcript for the UL146-UL132 locus, known to be upregulated by IE2, was significantly suppressed by treatment with N-methyl mesoporphyrin IX (NMM), a parallel G4-binding ligand. A putative G4-forming sequence, also called G4 motif, was identified in the UL146 promoter. Circular dichroism, fluorescence turn-on, and gel electrophoresis analyses confirmed the stable formation of a parallel G4 from this motif. In G4 pull-down assays, IE2 bound to G4, and NMM inhibited the G4 binding of IE2. In reporter assays, the G4 motif was necessary for activity of the UL146 promoter, and NMM treatment suppressed IE2-mediated transactivation of the UL146 promoter. UL146 transcription was significantly reduced in a recombinant virus with G4-disrupting mutations in the UL146 promoter, especially at low multiplicity of infection, along with decreased G4 formation and IE2 binding. Our findings demonstrate that G4 can act as a positive structural element in viral promoters, uncovering a novel mechanism of IE2-mediated transactivation.

**IMPORTANCE:**

G4-quadruplex (G4) is a noncanonical nucleic acid secondary structure that forms in single-stranded DNA or RNA. G4 structures are involved in various cellular processes, including transcription, translation, and DNA replication. It has been shown that G4 formation in gene promoters can inhibit promoter activation. Recently, a positive role of G4 in promoters has also been discovered, but evidence in viral promoters remains limited. In this study, we demonstrate that a G4 structure forms in the human cytomegalovirus UL146 promoter and acts as a binding site for the viral transactivator IE2, which plays a key role in viral gene expression. Using recombinant viruses, we confirm that IE2 binding to G4 is necessary for efficient UL146 transcription during virus infection. This study provides evidence that G4 structures in promoters can positively regulate viral gene expression and uncovers a novel mechanism by which IE2 activates gene expression.

## INTRODUCTION

G-quadruplexes (G4s) are noncanonical nucleic acid secondary structures formed by repetitive guanine-rich sequences in single-stranded DNA and RNA (1, 2). In a G4 structure, four guanine bases are connected by Hoogsteen hydrogen bonds, forming a G-tetrad, and multiple G-tetrads stack on top of each other. The stability of G4 is enhanced by cations such as K^+^ and Na^+^. G4s can be produced by a single molecule or multiple strands and adopt various topologies, including parallel, antiparallel, and hybrid conformations, depending on the sequence composition and environmental conditions (3). Since their initial discovery in telomeric regions, DNA or RNA G4s have been identified across genomes, as well as in coding and noncoding RNAs, playing regulatory roles in various cellular processes, including transcription, translation, DNA replication, and genome stability (3, 4).

Human cytomegalovirus (HCMV), a member of the betaherpesvirinae subfamily, is a common human pathogen with global seroprevalence estimates ranging from 60% to greater than 90%. HCMV establishes lifelong latency in hematopoietic progenitor and myeloid lineage cells. The reactivation of HCMV can lead to serious illness in immunocompromised individuals, including transplant recipients and newborns with congenital infection. The HCMV genome is approximately 235 kb in length and contains more than 200 open reading frames. Viral genes are expressed in a temporally regulated cascade of immediate-early (IE), E, and late gene expression that governs viral replication and host interaction (5).

Genome-wide studies have shown that HCMV contains many putative G4-forming sequences (PQSs), also known as G4 motifs, with a high frequency in repeated regions, gene promoters, and the lytic replication origin (6–8). G4s in promoters are known to suppress transcription by inhibiting the recruitment of transcription factors (9). We previously demonstrated that several G4 motifs are present in the HCMV promoter regions and can form stable G4 structures. Treatment with the G4 ligand N-methyl mesoporphyrin IX (NMM) suppressed several G4-containing promoters, indicating that G4 ligands suppress G4-containing viral promoters in a promoter context-dependent manner. By analyzing a G4 motif-deleted recombinant virus, we demonstrated that G4 formation in UL35 inhibits promoter activity and that the G4 ligand can suppress the promoter through G4 stabilization (7). Similarly, G4 formation in the HCMV miR-US33 promoter has been shown to suppress promoter activity (10).

Recently, it has also been shown that G4 formation can positively regulate promoter activity. G4s in the human genome are common binding hubs for various transcription factors and promote increased transcription (11). G4 formation in the cMyc oncogene promoter increases gene transcription (12). In herpes simplex virus type 1, the viral IE transcription factor ICP4 specifically binds to parallel-stranded G4 structures located in the IE gene promoters, enhancing their activation. Disruption of G4 formation impairs ICP4 recruitment and diminishes gene expression, supporting a role in activation (13). The promoters of viral Bcl-2 homologs, KS-Bcl-2 of Kaposi’s sarcoma-associated herpesvirus and BHRF1 of Epstein-Barr virus, contain G4 motifs. Treatment with the G4 ligand pyridostatin enhances the activity of KS-Bcl-2 and BHRF1 promoters, indicating a positive role for G4 formation in promoter activation (14). Recent integrative analyses of the human genome have demonstrated that promoter-associated G4s are subject to selective constraint and often correlate with high-level gene expression, supporting their stimulatory role in gene expression (15).

Although G4s have been linked to transcriptional regulation in the HCMV genome, their ability to positively regulate HCMV promoters remains underexplored. We recently demonstrated that the 86 kDa IE2 protein, a key regulator of HCMV gene expression and DNA replication (16, 17), directly binds to a parallel G4 in essential region I (ER-I) within the viral lytic origin of replication (*ori*Lyt), facilitating DNA replication (8). During HCMV infection, IE2 has been shown to transactivate several viral and cellular genes by interacting with general or specific transcription factors in host cells (18–20). Recent studies demonstrated that IE2 drives host RNA polymerase II (Pol II)-mediated transcription initiation in a subset of viral late infection promoters, either dependent or independent of viral late-acting transcription factors (LTFs) (21, 22). IE2 has also been shown to activate or repress transcriptional initiation and modulate Pol II elongation by directly binding to viral DNA (16).

The finding that IE2 interacts with a G4 led us to explore G4 targeting as a possible mechanism of IE2-mediated transactivation. In this study, we show that the UL146 promoter, significantly activated by IE2 during the late phase of infection independent of viral LTFs, is effectively suppressed by a parallel G4-binding ligand. We show that a parallel G4 is formed in the UL146 promoter and is directly targeted by IE2 and that this interaction is required for efficient activation of UL146 transcription during HCMV infection. This highlights a positive role for G4s in viral promoters and uncovers a novel G4-dependent mechanism of IE2-mediated transactivation.

## RESULTS

### Identification of the UL146-UL132 locus as an HCMV gene suppressed by NMM

**Fig 1:**
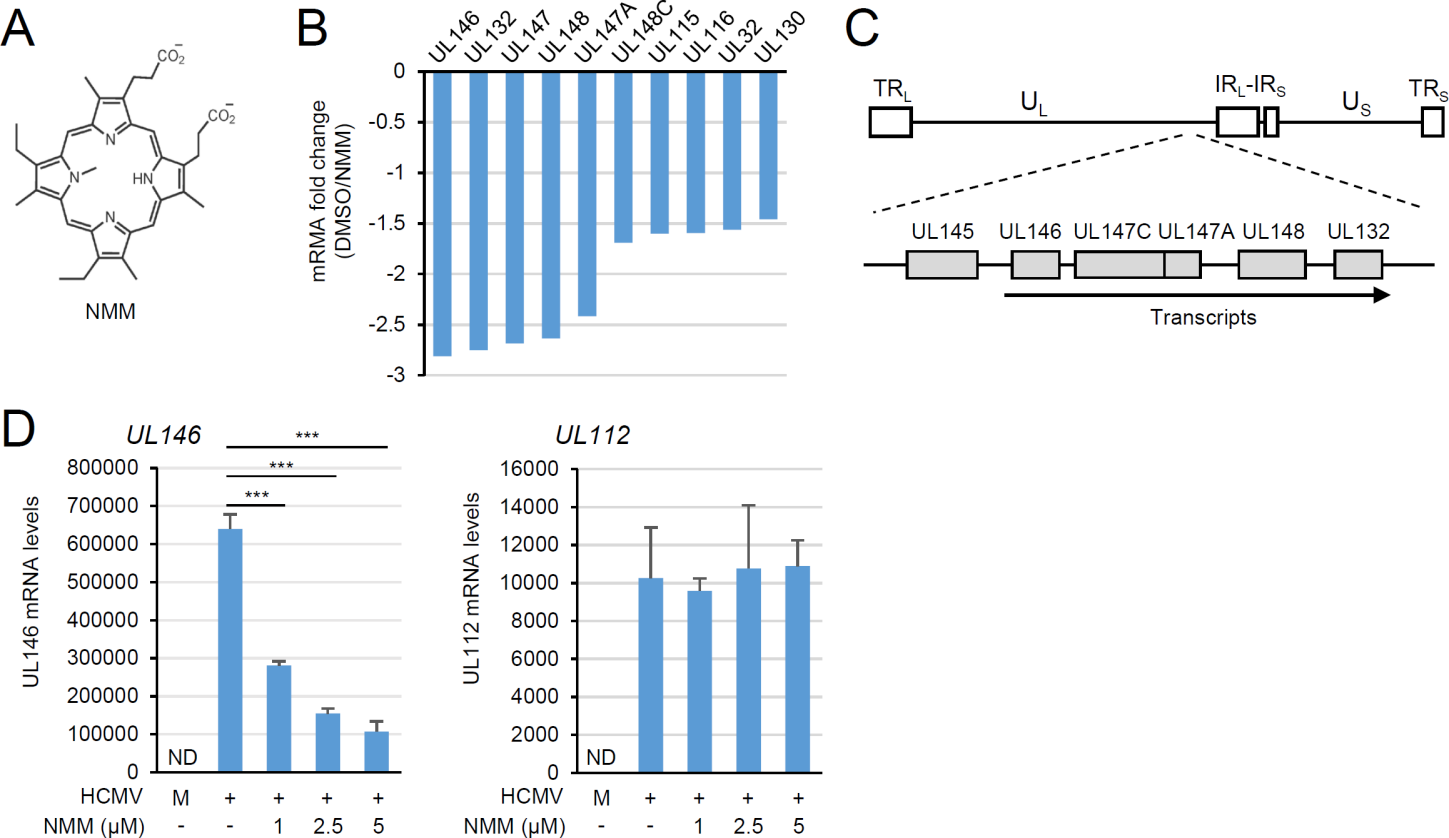
Suppression of the UL146 promoter by NMM treatment. (**A**) The chemical structure of NMM. (**B**) HF cells in 150 mm culture dishes were infected with HCMV at an MOI of 2. Cells were treated with DMSO or NMM (5 µM), incubated for 96 h, and subjected to RNA-seq analysis. The bar plot represents fold change in mRNA levels between DMSO-treated and NMM-treated cells. The top 10 downregulated genes are indicated. The data were obtained from a single experimental set. (**C**) The general structure of the HCMV genome (top) and the UL146-UL132 locus, where individual proteins are synthesized from a single transcript (bottom), are indicated. U_L_ (unique long), U_S_ (unique short), IR_L_ (internal repeat long), IR_S_ (internal repeat short), TR_L_ (terminal repeat long), and TR_S_ (terminal repeat short). (**D**) HF cells (2 × 10⁶ cells) in 12-well plates were mock-infected (M) or infected with HCMV at an MOI of 2. Cells were treated with DMSO (−) or increasing amounts of NMM as indicated, and total RNA was prepared at 96-h post-infection. The UL146 and UL112 mRNA levels were quantified by qRT-PCR with normalization to GAPDH. ND, not detected. Statistical significance is indicated by *P* < 0.001 (***).

We previously demonstrated that the addition of a G4 ligand, NMM, to HCMV (Toledo strain)-infected cells effectively suppressed the transcription of several viral genes and lowered the production of progeny virions (7). NMM is an asymmetric anionic porphyrin G4 ligand, which binds to G4 through π-π stacking and is highly specific to parallel G4 over antiparallel G4 and duplex DNA (23, 24) (Fig. 1A). To further investigate the effect of prolonged NMM treatment on the accumulation of viral transcripts, we infected human fibroblast (HF) cells with HCMV (Toledo strain) at a multiplicity of infection (MOI) of 5 and analyzed viral transcript accumulation at 96 h after infection using RNA-seq analysis. Although the RNA-seq data were obtained from a single experimental set, among the viral genes downregulated, UL146, UL132, UL147, UL148, and UL147A, which are expressed as a single transcript (25), were most effectively suppressed by NMM (Fig. 1B and C; File S1). Thus, NMM appeared to inhibit the transcription at the UL146-UL132 locus by affecting promoter activation. When we determined the effect of NMM on the viral mRNA levels in HCMV-infected cells by quantitative real-time PCR (qRT-PCR), NMM suppressed the accumulation of UL146 transcripts in a dose-dependent manner, while it did not affect the accumulation of UL112 transcripts, as seen in RNA-seq data (Fig. 1D). This indicates that NMM effectively suppresses transcription of the UL146-UL132 locus during virus infection.

### G4 formation in the HCMV UL146 promoter

**Fig 2:**
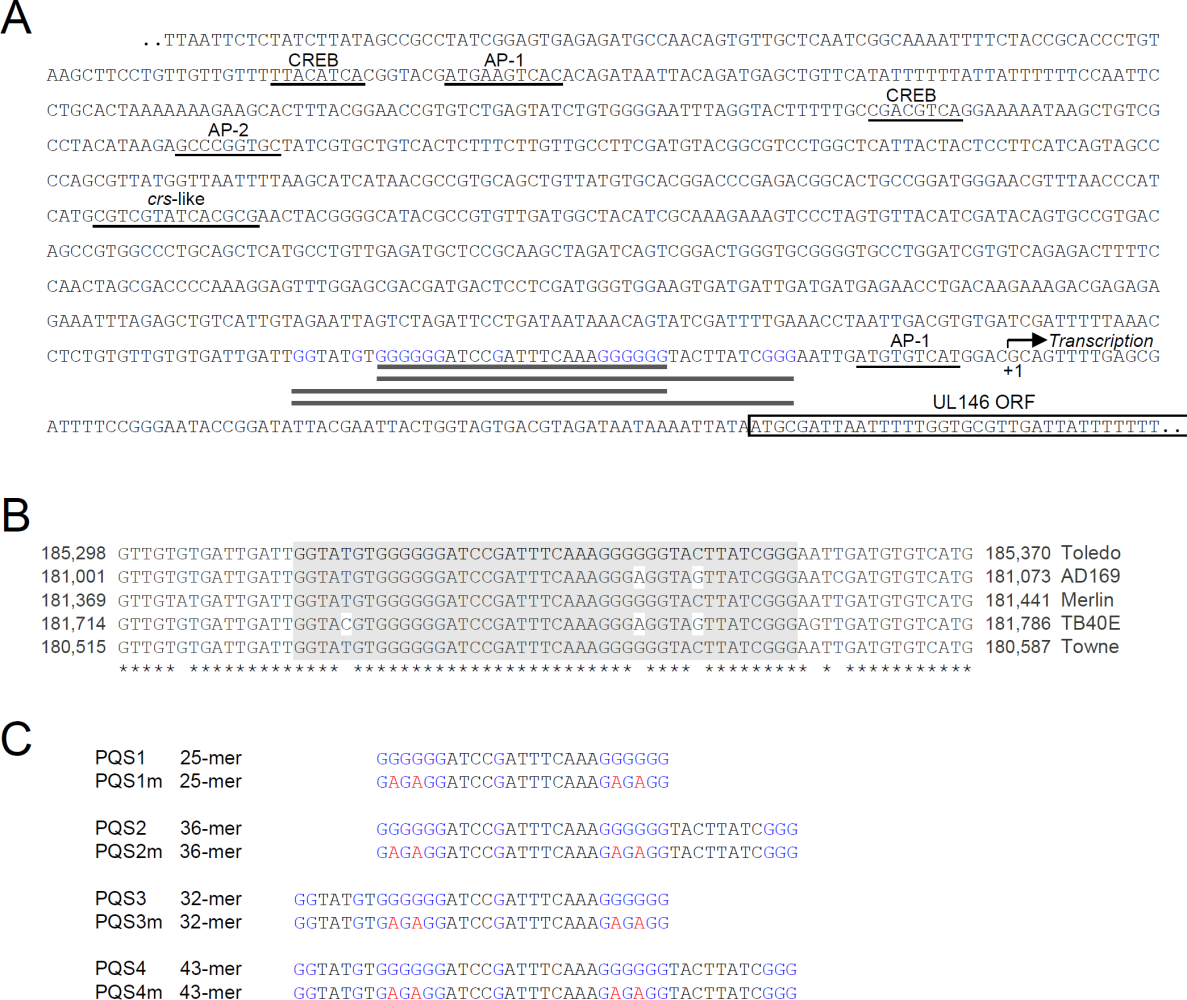
PQSs in the UL146 promoter. (**A**) The overlapping PQSs found in the UL146 promoter region (thick lines) and the positions of the UL146 transcription start site and the ORF are indicated. The *crs*-like site and the putative binding sites of cellular transcription factors known to interact with IE2 are also indicated (underlined). The cellular transcription factor binding sites were predicted using the JASPAR database (https://jaspar.elixir.no). Target sequences with a relative profile score above 90% are indicated. (**B**) The G4 motifs in the UL146 promoters of different HCMV strains were compared. The accession numbers of the viral genome sequences are GU937742.1 (Toledo), FJ527563.1 (AD169), AY446894.2 (Merlin), KF297339.1 (TB40E), and FJ616285.1 (Towne). (**C**) The PQS1-PQS4 sequences containing the G4 motif in (**A**) and their mutant sequences (PQS1m-PQS4m) used in the study are indicated. G-rich sequences in the G4 motif are indicated in blue, and G to A substitutions are indicated in red.

The UL146 promoter, which drives transcription of the UL146-UL132 locus during early-late kinetics, lacks a typical TATA box (25) or a TATT box required for transactivation by viral LTFs (21, 22). We identified a G-rich sequence from −61 to −19 bp upstream of the UL146 transcription start site (Fig. 2A). This G4 motif in the UL146 promoter is highly conserved in different HCMV strains (Fig. 2B). Several noncanonical PQSs, designated PQS1 to PQS4, were identified (Fig. 2C). To investigate G4 formation from these sequences, we synthesized the corresponding oligodeoxynucleotides (ODNs) and their mutant versions, where four guanines were replaced with adenines to disrupt G4-forming potentials (Fig. 2C). These were then used for circular dichroism (CD) analysis.

**Fig 3:**
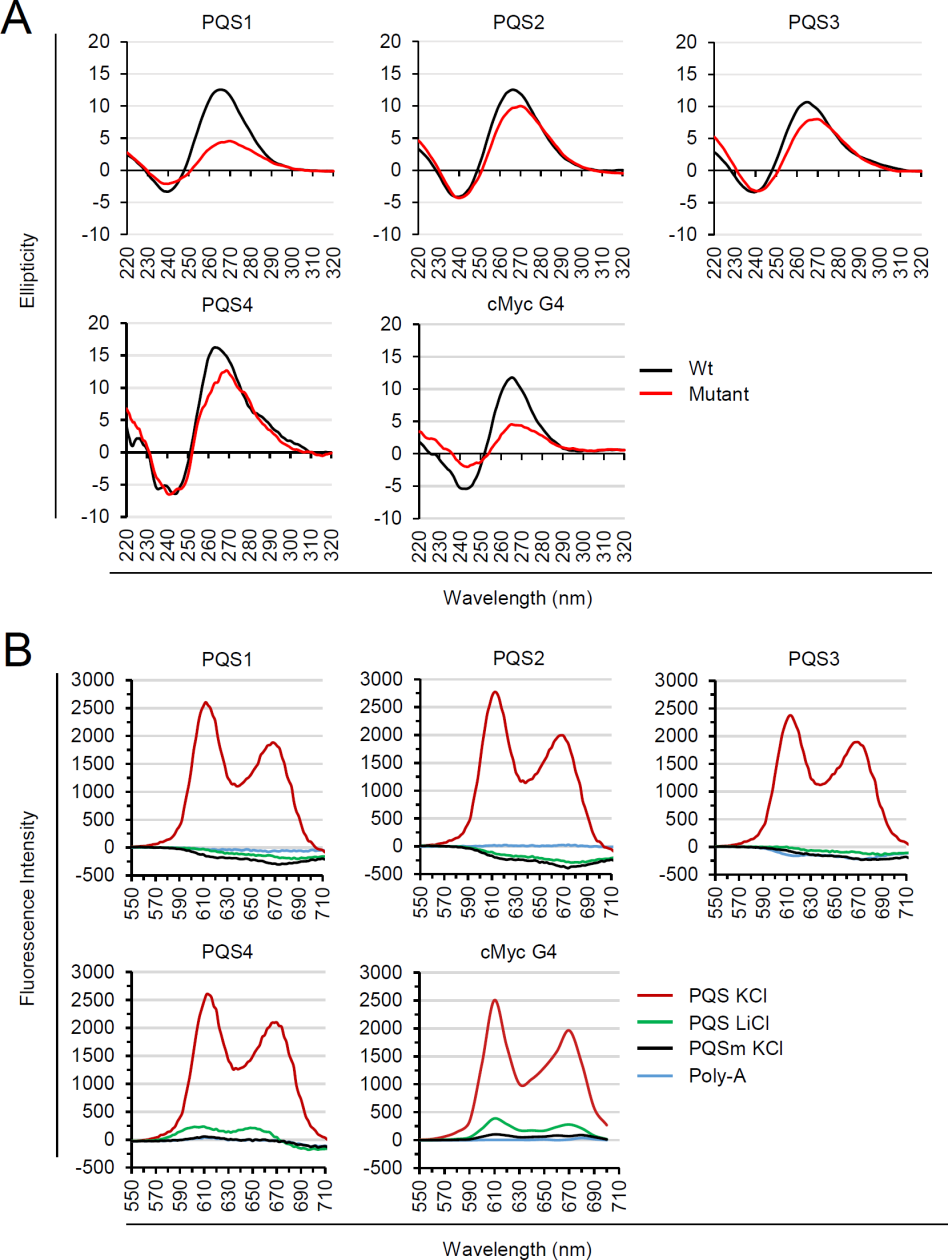
CD analysis and NMM fluorescence turn-on assay to determine G4 formation *in vitro*. (**A**) The CD spectra of wild-type and mutant UL146 PQS and cMyc-G4 ODNs (15 µM) annealed in 10 mM Tris-HCl (pH 7.5) and 100 mM KCl are shown. The spectral data were collected in the 220–320 nm range, with each spectrum representing the average of three scans. A buffer-only control was used for baseline correction. The data were normalized to the maximum ellipticity and processed using GraphPad Prism 5 for smoothing. (**B**) The preformed wild-type or mutant UL146 PQS and cMyc-G4 ODNs or poly-A (26-mer) ONDs were incubated with NMM (4 µM) in 100 mM KCl or LiCl for 20 min, followed by measurement of G4-bound NMM-specific fluorescence.

When the wild-type and mutant ODNs were incubated to form G4 structures and subjected to CD analysis, all the PQSs (PQS1 to PQS4) showed a positive peak at 265 nm and a negative peak at 240 nm, indicating the formation of a parallel G4. However, their mutants showed alteration in positive peak positions, suggesting reduced G4 formation. As controls, the wild-type and mutant cMyc G4 motifs exhibited similar patterns (Fig. 3A). NMM fluorescence intensity increases significantly upon binding to G4 relative to ssDNA (26). Consistent with the CD results, fluorescence turn-on assays with NMM showed that intact PQS1–PQS4, but not their mutants, displayed a typical G4-bound NMM fluorescence pattern under KCl buffer conditions, which stabilizes G4 structures, but not under LiCl conditions, where G4s are not formed. As a negative control, poly-A sequence failed to display the G4-bound NMM fluorescence pattern under KCl buffer conditions. Similar patterns were observed with the wild-type and mutant cMyc G4 motifs (Fig. 3B).

**Fig 4:**
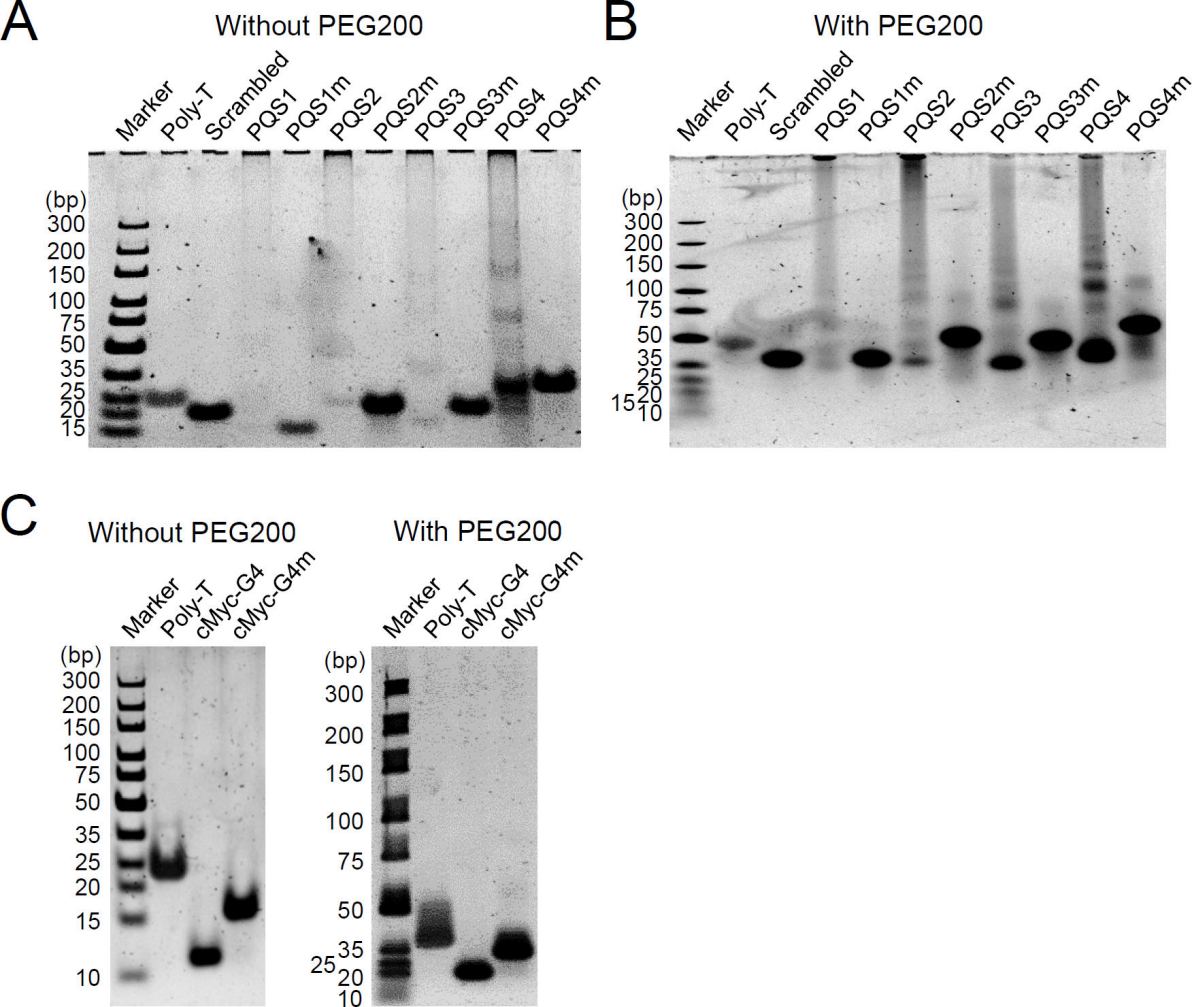
Native PAGE analysis to determine intra- and intermolecular G4 formation *in vitro*. (**A and B**) The wild-type and mutant UL146 PQS ODNs (5 µM) annealed in 100 mM K^+^ buffer were analyzed using 12% native PAGE without (**A**) or with (**B**) 40% (wt/vol) PEG200. Scrambled DNA (5′-TAACCGATGATATGAGTCAGATATAT-3′) and poly-T (26-mer) ODNs were used as controls. dsDNA size markers are shown. (**C**) Similar native PAGE analyses were performed with the wild-type and mutant cMyc-G4 motifs without or with 40% (wt/vol) PEG200, as indicated.

G4s can be formed intramolecularly in single-stranded DNA or intermolecularly by multiple strands. In native polyacrylamide gel electrophoresis (PAGE) analysis with G4-preformed wild-type and mutant ODNs, PQS1, PQS2, and PQS3 produced slowly migrating ladders, whereas their mutants yielded a band of unstructured single-stranded DNA. We reason that the weak signals for the wild-type sequences are due to the effective formation of intermolecular G4s. This indicates that PQS1, PQS2, and PQS3 favor the formation of intermolecular G4s *in vitro*. PQS4 formed both slowly migrating ladders and a faster migrating band, suggestive of intramolecular G4, in comparison to its mutant (Fig. 4A). The formation of intramolecular G4s from PQS2, PQS3, and PQS4 became apparent when G4s were formed under molecular crowding conditions with 40% (wt/vol) polyethylene glycol (PEG) 200 and analyzed by native PAGE, although the bands were shifted, probably due to PEG (Fig. 4B). As controls, the wild-type cMyc G4 motif also showed a faster migration pattern than its mutant version, cMyc-G4m (Fig. 4C). Collectively, the *in vitro* analyses results demonstrate that a parallel intramolecular G4 is formed in the UL146 promoter.

### NMM inhibits the binding of IE2 to UL146 G4

**Fig 5:**
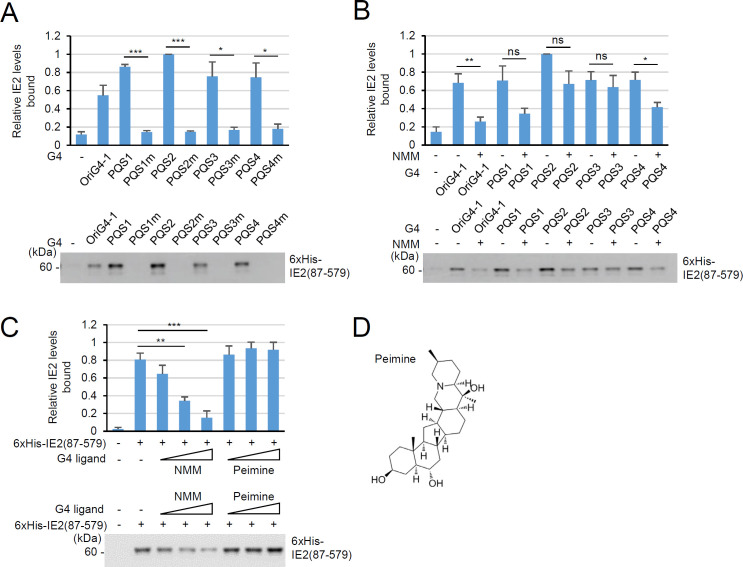
G4 pull-down assays demonstrating IE2 binding to UL146 G4 and the inhibitory effect of NMM. (**A**) G4 pull-down assays were conducted using purified 6 × His-IE2 (87–579). The biotinylated wild-type or mutant UL146 ODNs or oriG4-1 ODNs were preincubated to form structures and incubated with IE2, followed by pull-down assays (see Materials and Methods). The bound IE2 proteins were detected by immunoblotting using an anti-His antibody. (−), no ODNs. The relative binding efficiency of IE2 to ODNs is indicated in graphs (*n*  =  3). (**B**) G4 pull-down assays in (**A**) were performed with (+) or without (−) NMM treatment (DNA to chemical ratio 1:2). (**C**) G4 pull-down assays were conducted with PQS4 with or without treatment of increasing amounts of NMM or Peimine (DNA to chemical ratio from 1:1 to 1:4) as in (**B**). Statistical significance is indicated by *P* < 0.05 (*), <0.01 (**), and < 0.001 (***). (**D**) The chemical structure of Peimine. ns, not significant.

Since a G4 is formed in the UL146 promoter, which is thought to be effectively suppressed by NMM, and IE2 has parallel G4-binding activity (8), we examined whether IE2 binds to the UL146 promoter G4. Although the full-length IE2 was difficult to purify in bacterial cells due to low solubility, we could purify the N-terminal 86 amino acid-deleted IE2 form as a soluble protein. In G4 pull-down assays, IE2 (87–579) could bind to all G4s formed by PQS1 to PQS4 ODNs with a binding strength comparable to or greater than that formed by the oriG4-1 sequence in HCMV *ori*Lyt ER-I. However, it did not interact with mutant ODNs, indicating that IE2 directly binds to the UL146 promoter G4 (Fig. 5A).

Given that NMM suppressed UL146 transcription, we tested whether NMM can interfere with IE2 binding to the UL146 promoter G4. In G4 pull-down assays, NMM inhibited IE2 binding to the G4s formed by PQS1, PQS2 (though not statistically significant), and PQS4, while it did not affect IE2 binding to the G4 formed by PQS3 (Fig. 5B). PQS4m showed a significant reduction in IE2 binding by NMM treatment. In addition, PQS4, longer than other PQSs, formed an intramolecular G4 effectively. Therefore, we used PQS4 for further analysis of IE2 binding. Unlike NMM, which binds to a parallel G4 through a G-quartet (26), the G4 groove binder Peimine (dihydroisoimperialine) (27) did not inhibit the binding of IE2 to the G4 (Fig. 5C and D). This indicates that IE2 binding to UL146 G4 may involve G-quartet binding. Collectively, these results demonstrate that the ability of IE2 to bind UL146 G4 can be inhibited by NMM.

### IE2 transactivates the UL146 promoter through G4

**Fig 6:**
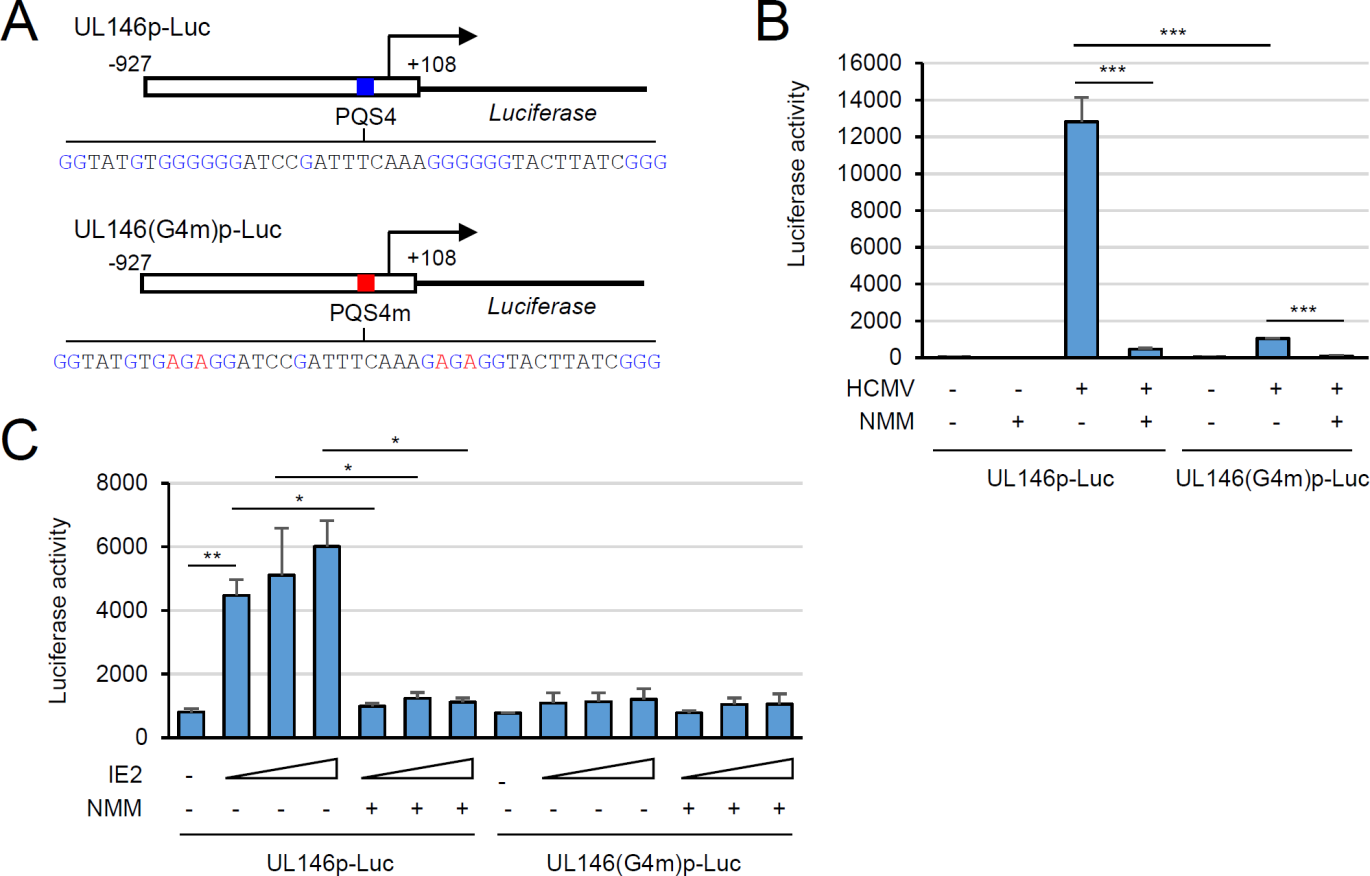
Requirement of G4 formation for UL146 promoter activation in reporter assays. (**A**) Diagrams illustrating the luciferase reporter genes driven by the wild-type UL146 promoter (UL146p) or the G4 formation-defective promoter (UL146(G4m)*P*) containing PQS4m. G-rich sequences in the PQS4 are indicated in blue, and G to A substitutions in PQS4m are indicated in red. Numbers in the UL146 promoter region are indicated with the UL146 transcription start site as + 1. (**B**) HF cells (2.5 × 10^5^ cells) were electroporated with 1 µg of UL146p-Luc or UL146(G4m)-Luc reporter plasmid and plated in 12-well plates. Three days after electroporation, cells were infected with HCMV at an MOI of 2 and incubated with or without NMM (5 µM). At 72-h post-infection, luciferase activity was measured. Statistical significance is indicated by *P* < 0.001 (***). (**C**) U373-MG cells (2.5 × 10^5^ cells) in 12-well plates were co-transfected with a plasmid containing the wild-type or G4 mutant reporter gene (0.5 µg) and increasing amounts (0.2, 0.4, or 0.8 µg) of plasmids expressing IE2, as indicated. At 6 h after transfection, the medium was replaced with fresh medium with or without NMM or Peimine (5 µM). Cells were further incubated for 18 h, and then luciferase activity was measured. Statistical significance is indicated by *P* < 0.05 (*), <0.01 (**), and < 0.001 (***).

We next examined whether IE2 transactivates the UL146 promoter by targeting G4. To achieve this, we created reporter plasmids containing the luciferase reporter gene driven by the intact UL146 promoter (UL146p) or the G4-defective mutant promoter (UL146(G4m)p), in which the PQS4 sequence was replaced with the PQS4m sequence (Fig. 6A). HF cells were transfected with these reporter plasmids via electroporation and then infected with HCMV, with or without NMM treatment, followed by luciferase assays. The UL146(G4m) promoter had a 12-fold reduction in activity compared to the wild-type promoter in virus-infected cells, indicating that the G4 motif is essential for efficient UL146 promoter activation (Fig. 6B). We also found that NMM significantly suppressed the UL146 promoter activity by 27-fold, while it reduced the activity of the UL146(G4m) promoter by 10-fold (Fig. 6B). These findings suggest that NMM primarily inhibits the UL146 promoter through G4, although it can also suppress promoter activity in a G4-independent manner, likely by downregulation of other viral genes necessary for UL146 promoter activation.

To assess the effect of NMM on IE2-mediated UL146 promoter activation, we also conducted co-transfection reporter assays in U373-MG cells, which are semi-permissive to HCMV infection and exhibit high transfection efficiency. We observed that IE2 alone could transactivate the UL146 promoter but not the UL146(G4m) promoter and that NMM effectively suppressed the UL146 promoter activated by IE2 (Fig. 6C). These findings demonstrate that IE2 transactivates the UL146 promoter through G4, and NMM interferes with IE2-mediated UL146 transactivation.

### Evaluation of G4-dependent IE2 transactivation of the UL146 promoter using recombinant viruses

**Fig 7:**
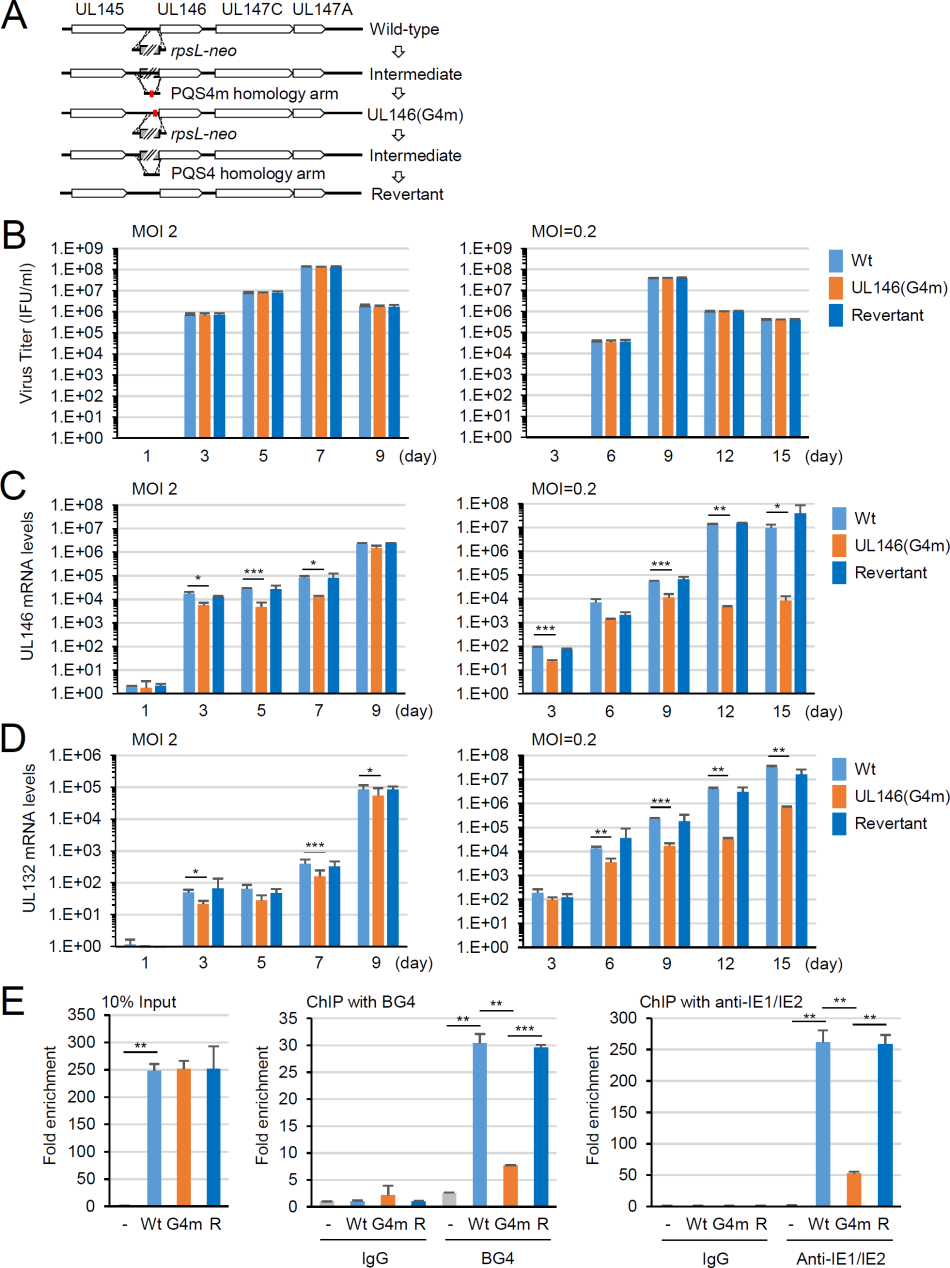
Analysis of recombinant viruses containing G4-disrupting mutations in the UL146 promoter. (**A**) Scheme for bacmid mutagenesis (see Materials and Methods). (**B–D**) HF cells were infected with recombinant viruses containing the wild-type UL146 promoter (Wt), G4-defective UL146 promoter (UL146(G4m)), or its revertant promoter at an MOI of 2 or 0.2. The culture supernatants were collected at 1, 3, 5, 7, or 9 days after infection (for MOI of 2) or at 3, 5, 7, 9, 12 days (for MOI of 0.2), and viral titers (IFU/mL) were determined by infectious center assays (**B**). The cells were harvested at the indicated time points, and the mRNA levels of UL146 (**C**) and UL132 (**D**) were determined by qRT-PCR and normalized to GAPDH. (**E**) HF cells were infected with recombinant viruses at an MOI of 2 for 4 days, followed by ChIP assays with BG4 antibody, anti-IE1/IE2 antibody, or control IgG. The ChIP samples were subjected to qPCR with the primer sets that amplified the G4 motif-containing UL146 promoter region. For the ChIP results, the fold enrichment of PCR products over those with empty vector control (for 10% input) or IgG in mock infection (for ChIP) is shown. The data are presented as the mean ± SD (*n* = 3). Statistical significance is indicated by *P* < 0.05 (*), <0.01 (**), and < 0.001 (***).

Activation of the UL146 promoter by IE2 through G4 was further examined by producing a recombinant HCMV (UL146(G4m)) with G4-disrupting mutations (PQS4m sequence) in the UL146 promoter, along with its revertant virus. The HCMV (Toledo strain) bacmids containing PQS4m and its revertant were produced by bacmid mutagenesis using the counter-selection marker *rpsL-neo* (Fig. 7A). The recombinant viruses were grown in HF cells that received the bacmid DNA via electroporation. UL146 encodes a viral chemokine, vCXCL1, which is not essential for viral growth in cell culture (28, 29). Consistently, the UL146(G4m) virus exhibited a growth curve similar to those of the wild-type and revertant viruses at a MOI of 2 or 0.2 (Fig. 7B). However, the UL146 transcript levels measured by real-time quantitative PCR (RT-qPCR) were about sixfold lower in UL146(G4m) virus infection at 5 or 7 days post-infection than in wild-type and revertant virus infections at an MOI of 2. At an MOI of 0.2, this reduction was more pronounced to approximately 3,000-fold at 12 days (Fig. 7C). Similarly, the UL132 mRNA levels were about 2.5-fold and 124-fold lower at MOIs of 2 (7 days) and 0.2 (12 days), respectively, during UL146(G4m) virus infection (Fig. 7D). These results indicate that G4 formation in the UL146 promoter is necessary for efficient transcription of the UL146-UL132 locus during virus infection, especially at low MOI.

To confirm G4 formation in the UL146 promoter and IE2 binding to G4 during virus infection, HF cells were infected with recombinant viruses, and chromatin immunoprecipitation (ChIP) assays were conducted using antibodies for G4 and IE2. Since the IE2-specific antibody we used was ineffective for ChIP assays, we used an antibody that detects the common N-terminal region of IE1 and IE2. We observed that G4 formation in the UL146 promoter region and the IE2 protein level associated with the promoter were significantly lower in the UL146(G4m) virus infection compared to wild-type and revertant virus infections (Fig. 7D). These findings with recombinant viruses demonstrate that IE2 transactivates the UL146 promoter through G4 during virus infection.

## DISCUSSION

In this study, we demonstrate that HCMV IE2 transactivates the UL146 promoter by targeting a G4 structure located immediately upstream of the transcription start site. The G4-forming sequence identified in the UL146 promoter was not predicted in our previous genome-wide search for G4 motifs, in which we used a G_(3–6)_N_(1–7)_G_(3–6)_N_(1–7)_G_(3–6)_N_(1–7)_G_(3–6)_ schema (7). However, the findings that UL146 transcription depends on IE2 (21, 22); that IE2 binds to a parallel G4; and that NMM, a parallel G4-binding ligand, effectively suppresses UL146 transcription (this study), led us to investigate potential G4 formation within the UL146 promoter. We found a noncanonical G4 motif in the UL146 promoter and confirmed the formation of a parallel G4 structure by analyzing the corresponding ODN in CD analysis, G4-ligand fluorescence turn-on assay, and native PAGE *in vitro*. Additionally, G4 formation in the viral genome during infection was validated by G4-ChIP assays using a G4-specific antibody in recombinant virus-infected cells. It remains unclear whether the G4 formed in the UL146 promoter has a structure with two G-quartets or a bulged structure with three G-quartets. However, our native PAGE analysis showed that the G4 motif can adopt an intramolecular G4 structure, which may be important for transcriptional control in the viral genome during infection.

We demonstrated that IE2 binds directly to the UL146 promoter G4 *in vitro*. The strength of IE2 binding to this G4 was comparable to that of the *ori*Lyt ER-I G4. Consistent with its inhibitory effect on IE2-mediated UL146 transcription during virus infection, NMM inhibited IE2 binding to the UL146 promoter G4. Interestingly, NMM inhibited IE2 binding to the G4 formed by PQS1, 2, and 4 but not that by PQS3, although IE2 bound to the G4s formed by all PQS ODNs. This suggests that the G4 structure formed by PQS3 may differ from those formed by the other ODNs, and the G4-binding modes of IE2 are also different. We previously demonstrated that, when IE2 binds to a parallel G4 structure in *ori*Lyt ER-I, G-quartet binding is more important than groove binding (8). IE2 seems to recognize UL146 G4 in a similar way, because Peimine, which binds to G4 grooves, did not inhibit its binding to UL146 G4. TMPyP4 (tetra(*N*-methyl-4-pyridyl)porphyrin) is a potent porphyrin G4 binder with a 4^+^ charge and a planar core that enables interaction with G4 through electrostatic interactions (30), although it shows low selectivity for G4 versus double-stranded DNA (dsDNA) (31). Indeed, TMPyP4 was more effective at inhibiting the binding of IE2 to *ori*Lyt ER-I G4 than NMM (8). When we tested the effect of TMPyP4 on IE2 binding to UL146 G4 and IE2-mediated transactivation of the UL146 promoter, TMPyP4 more effectively inhibited the UL146 G4 binding by IE2, but its suppression of IE2-mediated UL146 promoter activation was similar to that of NMM (S. Gong and J.-H Ahn, unpublished data). We deduce that, although NMM has relatively low affinity for G4 because of its negative charge and nonplanarity, it functions as a good G4 binder in cells, likely due to its efficient translocation.

Our findings that IE2 transactivates the UL146 promoter by targeting a promoter G4 structure support the view of G4 as a positive structural element that regulates promoter activity by acting as a binding site for transcription factors (11, 15). We previously showed that treatment with G4 ligands suppresses G4-containing HCMV promoters in a context-dependent manner (7). This suppressive effect can be explained in two ways. G4 can act as a negative element for promoter activation by inhibiting the RNA Pol II complex, and G4 stabilization by ligands further suppresses promoter activation. Conversely, G4 can be a positive element that recruits transcription factors. However, G4 ligands may interfere with the G4 binding of transcription factors. The results of the current study provide evidence that G4 can act as a positive element in promoter activation in the HCMV genome. Using genome-wide functional studies of a specific G4 structure with recombinant viruses, we demonstrated G4 formation in the UL146 promoter and its binding to IE2. We also demonstrated the positive role of G4 binding by IE2 in UL146 transcription during virus infection. Interestingly, we found that the mutant virus containing the UL146 promoter lacking G4-forming potential exhibited a more severe reduction of UL146 transcription at low MOI than at high MOI. This finding indicates that UL146 promoter activation mainly depends on G4 targeting by IE2 at low MOI but may also involve G4-independent mechanisms at high MOI. IE2 binds to a 14 bp *cis-r*epression *s*equence (*crs*), a CG dinucleotide-flanked AT-rich sequence, within the major IE promoter, repressing its transcription through negative feedback regulation (32–37). The binding of IE2 to the *crs*-like site appears to be involved in the activation of some viral promoters by IE2 (16, 38–40). We found one *crs*-like site and several putative binding sites of alkaline phosphatase (AP)-1, AP-2, and CREB, which are known to interact with IE2, in the UL146 promoter region (Fig. 2A). IE2 could not activate the G4 motif-disrupted UL146 promoter in our reporter assays with co-transfected cells. However, it cannot be ruled out that the presence of these sites contributes to the significant activation of the G4-less UL146 promoter during high MOI infection. IE2 has been shown to bind the *crs* as a dimer or oligomer *in vitro* (41, 42). Whether IE2 binds to G4 as a monomer, dimer, or oligomer remains to be addressed. It would be intriguing to assess whether different states of IE2 multimerization influence its binding to different nucleic acid structures in the viral genome.

IE2 has also been shown to bind directly to several viral early promoters and cooperate with cellular transcriptional factors in activating transcription (39, 40, 43, 44). A recent study on genome-wide IE2 occupancy has demonstrated that IE2 binds to duplex DNA near core promoter regions to activate many early-late and late promoters (16). Our study for the first time provides evidence that IE2 is also able to transactivate a viral early-late promoter that lacks an upstream TATA or TATT sequence by directly binding to a G4 structure formed in the promoter. This work underscores the different modes of IE2 association with the viral genome and the importance of G4 structures in HCMV gene regulation. Given that G4-binding ligands can effectively inhibit the binding of IE2 to G4, our study also highlights these compounds as promising antivirals capable of disrupting the activation of select IE2-dependent G4-containing viral and cellular promoters.

## MATERIALS AND METHODS

### Cell culture, virus, and chemicals

Primary HF cells and human glioblastoma U373-MG cells (ATCC) were cultured in Dulbecco’s modified Eagle’s medium supplemented with 10% fetal bovine serum, 100 U/mL penicillin, and 100 µg/mL streptomycin. The cells were maintained at 37°C in a humidified incubator with 5% CO₂. The recombinant HCMV (Toledo strain) was grown in HF cells that received bacmid DNA as previously described (45). NMM was purchased from Santa Cruz Biotechnology. TMPyP4 was purchased from GLPbio. Peimine was obtained from Cayman Chemical.

### Plasmids, transfection, and electroporation

pMP18 expressing IE2 was previously described (46). To produce the UL146 promoter-driven luciferase reporter plasmid (UL146p-Luc), the UL146 promoter region (−927 to +108) was PCR-amplified as an NheI/NcoI fragment and cloned into pGL3-basic (Addgene). UL146(G4m)p-Luc containing the PQS4m sequence was produced by site-directed mutagenesis. U373-MG cells were transfected using polyethylenimine (Sigma-Aldrich). HF cells were transiently transfected via electroporation at 1,700 V for 20 ms using a Microporator MP-100 (Digital Bio) according to the manufacturer’s instructions.

### Infectious center assay

Virus titers were assessed using infectious center assays. HF cells (1 × 10⁵ cells) were plated in 24-well plates and incubated for 24 h before infection. Serial dilutions (10-fold) of viral stocks (200 µL per well) were added and adsorbed to cells for 1 h. The inoculum was then removed, and 1 mL of fresh complete medium was added, followed by an additional 24-h incubation. Cells were fixed with 500 µL methanol at −20°C for 10 min, washed three times with cold phosphate buffered saline (PBS), and incubated with 200 µL of anti-IE1 rabbit polyclonal antibody in PBS at 37°C for 1 h. Subsequently, the cells were treated with a phosphatase-labeled anti-rabbit IgG antibody under the same conditions. For visualization, 200 µL of AP buffer (100 mM Tris-HCl, 100 mM NaCl, and 5 mM MgCl₂) was mixed with BCIP/NBT substrate (1:1 ratio, Millipore) and applied to the cells. IE1-positive cells were counted in five fields per well using a light microscope at 200 × magnification.

### RNA-seq analysis

Total RNA was extracted using TRIzol Reagent (Thermo Fisher Scientific) and treated with DNase. For total RNA-seq, ribosomal RNA was depleted (Ribo-Zero rRNA removal), and the remaining RNA was randomly fragmented and reverse-transcribed into cDNA, followed by ligation with Illumina adapters, PCR amplification, and size selection to obtain ~200–400 bp inserts. Libraries were prepared with the Illumina TruSeq Stranded Total RNA LT Sample Prep Kit (Illumina) and sequenced by Macrogen on an Illumina platform (paired-end, 101 bp reads).

### CD spectroscopy

ODNs were dissolved at a concentration of 15 µM in buffer containing 10 mM Tris-HCl (pH 7.5) and either 100 mM KCl or 100 mM LiCl. The samples were heated to 95°C for 5 min to denature, cooled, and annealed overnight at room temperature. CD spectra were acquired at 25°C using a Jasco J-810 spectropolarimeter equipped with a Peltier temperature controller. Measurements were performed from 220 to 320 nm, averaging three accumulations, with a response time of 1 s, a scanning speed of 100 nm/min, and a data pitch of 1 nm. The wild-type and mutant cMyc-G4 motifs were used as controls: 5′-TGAGGGTGGGTAGGGTGGGTAA-3′ (22-mer, for cMyc-G4) and 5′-TGAGAGTGAGTAGAGTGAGTAA-3′ (22-mer for cMyc-G4m).

### NMM fluorescence turn-on assay

ODNs were prepared at a final concentration of 2 µM in G4 folding buffer containing 10 mM Tris-HCl (pH 7.5) and 100 mM KCl. Samples were heated at 95°C for 5 min to denature secondary structures and then slowly cooled to room temperature overnight. NMM was then added to each sample at a final concentration of 4 µM, followed by incubation at room temperature in the dark for 10 min to allow binding. Fluorescence emission spectra were recorded using a BioTek Synergy Neo (Agilent) spectrofluorometer with excitation at 394 nm and emission collected from 550 to 750 nm. The slit widths for both excitation and emission were set to 2 nm.

### Native PAGE

ODNs (5 µM) were dissolved in 10 mM Tris-HCl buffer (pH 7.5, room temperature), denatured by heating at 95°C for 5 min, and then cooled overnight at room temperature. The samples were analyzed via native 12% PAGE with or without 40% (wt/vol) PEG 200. The gel was stained with SYBR Gold and scanned using a GelDoc XR+ System (Bio-Rad).

### G4 pull-down assay

Biotin-tagged ODNs (11.3 µM) preincubated to form structures were incubated with streptavidin agarose beads (30 µg, Sigma S1638) in buffer (10 mM Tris, pH 7.5, 1 mM EDTA, 1 M NaCl, and 0.003% NP40) at room temperature for 30 min with rotation. After centrifugation, the immobilized ODNs were blocked with bovine serum albumin-containing buffer to minimize nonspecific interactions. ODNs were then incubated at 4°C for 1 h with bacterially purified IE2 proteins (2 µg) in binding buffer. After three washes, the ODN/protein complexes were resuspended in SDS-PAGE sample buffer, incubated at 37°C for 15 min, and then boiled at 97°C for 7 min, followed by SDS-PAGE and immunoblot analysis.

### Real-time qPCR

Total RNA was extracted from the cells using TRIzol Reagent (Thermo Fisher Scientific) and MaxTract High Density (Qiagen). A QuantiTect Reverse Transcription kit (Qiagen) was used to generate cDNA. RT-qPCR was conducted using Power SYBR Green PCR Master Mix and a QuantStudio Real-Time PCR System. The primers used to amplify the UL146 mRNA were 5′-ATAAGCGGGAGATGTGGATG-3′ (forward) and 5′-ACATTAAATATTATCCTCTAACACCTA-3′ (reverse). The primers used for the GAPDH mRNA were 5′-AAATCCCATCACCATCTTCCA-3′ (forward) and 5′-AGGGGCCATCCACAGTCTTCT-3′ (reverse).

### Luciferase reporter assay

Cells were collected and lysed through three freeze-thaw cycles in 100 µL of 0.25 M Tris-HCl (pH 7.9) with 1 mM dithiothreitol. After centrifugation to clarify the extracts, 30 µL of cell extract was incubated with 350 µL of reaction buffer A (25 mM glycylglycine [pH 7.8], 15 mM ATP, and 4 mM EGTA). The mixture was then combined with 100 µL of 0.25 mM luciferin (Sigma-Aldrich) in reaction buffer A. Luciferase activity was measured using a TD-20/20 luminometer (Turner Designs) over a 10-s assay, recorded in relative light units.

### HCMV bacmid mutagenesis

UL146 mutant and revertant Toledo-bacmids were generated using the Red/ET recombination system with a BAC Modification Kit (Gene Bridges). Briefly, an *rpsL-neo* selection cassette flanked by 100-nucleotide homology arms corresponding to the regions upstream and downstream of the UL146 target site was amplified by PCR. The resulting PCR products were purified and introduced into *Escherichia coli* DH10B cells harboring the wild-type HCMV (Toledo)-bacmid by electroporation (Gene Pulser II, Bio-Rad). Transformants containing the cassette were selected on Luria-Bertani (LB) agar plates supplemented with kanamycin (50 µg/mL). To generate the desired UL146 mutations, two complementary single-stranded ODNs encoding the mutant sequence were annealed to form dsDNA fragments, which were then introduced by homologous recombination to replace the *rpsL-neo* cassette. Recombinant colonies were selected on LB agar plates containing streptomycin (100 µg/mL), and correct integration was confirmed by PCR amplification and direct sequencing of the UL146 region. To generate the revertant bacmid, the *rpsL-neo* cassette was re-inserted at the UL146 locus of the mutant bacmid, followed by replacement with annealed ODNs containing the wild-type UL146 sequence using the same recombination strategy described above. All modified bacmids were verified by sequencing before use.

### ChIP assay

The ChIP Assay Kit (Millipore, #17-295) was used according to the manufacturer’s instructions. HF cells (2 × 10⁶ cells) infected with HCMV were harvested at 4 days post-infection. Cells were cross-linked with 1% formaldehyde for 10 min at room temperature and then lysed using the buffer provided in the kit. Chromatin was sheared by sonication to an average size of 200–600 bp. Immunoprecipitations were carried out using G4-specific BG4 antibody, anti-IE1/IE2 antibody, or control IgG. After immunoprecipitation and washing, cross-links were reversed, and DNA was purified. The immunoprecipitated DNA was analyzed by qPCR using primer sets amplifying the G4-containing UL146 promoter region. The primers for UL146 were 5′-ATCGATTTTGAAACCTAATTGA-3′ (forward) and 5′-TACCAGTAATTCGTAATATC-3′ (reverse). PCR amplification was performed under the following conditions: 95°C for 5 min, followed by 50 cycles of 95°C for 30 s and 55°C for 30 s.

### Statistical analysis

Samples were compared using Student’s *t*-test, and significance was defined as *P*  <  0.05 (*P  <  0.05, **P  <  0.01, or ***P  <  0.001).

## Data Availability

All data supporting the findings of this study are included in the article and its supplemental material and are also available from the corresponding author upon request.

## SUPPLEMENTAL MATERIAL

Supplemental material jvi.01467-25-s0001.xlsx Raw RNA-seq data.

ASM does not own the copyrights to Supplemental Material that may be linked to, or accessed through, an article. The authors have granted ASM a non-exclusive, world-wide license to publish the Supplemental Material files. Please contact the corresponding author directly for reuse.
