# Erratum for Gong et al., “Evidence for G-quadruplex-mediated transactivation by the immediate-early 2 protein of human cytomegalovirus”

**DOI:** 10.1128/jvi.00880-26

**Published:** 2026-06-18

**Authors:** Shuang Gong, Daegyu Park, Woo-Chang Chung, Jin-Hyun Ahn

## ERRATUM

Volume 99, no. 12, e01467-25, 2025, https://doi.org/10.1128/jvi.01467-25. Discussion, 3rd paragraph: “alkaline phosphatase (AP)-1” should read “activator protein 1 (AP-1).”

